# The impact of central line insertion bundle on central line-associated bloodstream infection

**DOI:** 10.1186/1471-2334-14-356

**Published:** 2014-07-01

**Authors:** Hung-Jen Tang, Hsin-Lan Lin, Yu-Hsiu Lin, Pak-On Leung, Yin-Ching Chuang, Chih-Cheng Lai

**Affiliations:** 1 Department of Medicine, Chi Mei Medical Center, Tainan, Taiwan; 2 Department of Health and Nutrition, Chia Nan University of Pharmacy and Science, Tainan, Taiwan; 3 Department of Nursing, Chi Mei Medical Center, Liouying, Tainan, Taiwan; 4 Department of Nursing, Min-Hwei College of Health Care Management, Tainan, Taiwan; 5 The Committee of Infection Control, Chi Mei Medical Center, Liouying, Tainan, Taiwan; 6 Department of Medical Research, Chi Mei Medical Center, Tainan, Taiwan; 7 Department of Internal Medicine, Chi Mei Medical Center, Liouying, Tainan, Taiwan; 8 Department of Intensive Care Medicine, Chi Mei Medical Center, Liouying, Tainan, Taiwan

**Keywords:** Central line bundle, Central line-associated bloodstream infection, Intensivist

## Abstract

**Background:**

Knowledge about the impact of each central line insertion bundle on central line-associated bloodstream infection (CLABSI) is limited.

**Methods:**

A quality-improvement intervention, including education, central venous catheter (CVC) insertion bundle, process and outcome surveillance, have been introduced since March 2013. Outcome surveillances, including CLABSI per 1,000 catheter-days, CLABSI per 1,000 inpatient-days, and catheter utilization rates (days of catheter use divided by total inpatient-days), were measured. As a baseline measurement for a comparison, we retrospectively collected data from March 1, 2012 to December 31, 2012.

**Results:**

During this 10-month period, there were a total of 687 CVC insertions, and 627 (91.2%) insertions were performed by intensivists. The rate of CLABSI significantly declined from 1.65 per 1000 catheter-day during the pre-intervention period to 0.65 per 1000 catheter-day post-intervention period (*P* = 0.039). CLABSI more likely developed in subjects in which a maximal sterile barrier was not used compared with subjects in which it was used (*P* = 0.03). Moreover, CVC inserted by non-intensivists were more likely to become infected than CVC inserted by intensivists (*P* = 0.010).

**Conclusions:**

This multidisciplinary infection control intervention, including a central line insertion care bundle, can effectively reduce the rate of CLABSI. The impact of different care bundle varies, and a maximal sterile barrier precaution during catheter insertion is an essential component of the care line insertion bundle.

## Background

In conjunction with the increasing use of central venous catheters (CVC) among critically ill patients, the occurrence of central line-associated bloodstream infections (CLABSI) is increasing. Recent studies have shown that this serious complication could result in increasing mortality, morbidity and hospital stay length [1-6]. Therefore, several evidence-based interventions, including the use of chlorhexidine gluconate (CHG) skin preparations and maximal sterile barriers during insertion, use of the subclavian or internal jugular vein instead of the femoral vein, hand hygiene, and daily review of line necessity, were developed to prevent CLABSI [7-10]. Moreover, these strategies were compiled into a “central line bundle” by the Institute for Healthcare Improvement (IHI).

To reduce the CLABSI rate in intensive care units (ICU), we introduced a multidimensional program, which included the implementation of central line bundle, education and surveillance investigations, in five adult ICUs in a regional hospital in southern Taiwan. The aim of this study was to evaluate the different impacts of each bundle on the ICU CLABSI rate from March 2013 to December 2013. To clarify the overall effect of this multidisciplinary team care bundle, we used the rate of CLABSI in the same period (from March to December) in 2012 as reference for the purpose of comparison.

## Methods

### Setting

This study was conducted in five adult ICUs at a regional teaching hospital, which had 63 ICU beds (including 26 beds for surgical ICU, 23 for medical ICU, and 14 for cardiac care unit) and eight intensivists. Beginning in March 2013, a quality-improvement intervention, including education, CVC insertion bundle, and process and outcome surveillance, were introduced in the ICU. In March, all ICU members, including physicians and nurses, were educated about the scope and practice of each central line bundle. The education program included the three times of lectures for all ICU personnel and the creation of teaching video which provided instruction for site selection, skin preparation, draping, insertion and dressing the central venous catheter. All of ICU members were asked to watch the video. The insertion bundle included four components: hand hygiene, maximal sterile barriers upon insertion, use of CHG for skin preparations, and avoidance of the femoral vein as the access site [11]. The maintenance bundle included hand hygiene, proper dressing changes, aseptic technique for accessing and changing needleless connectors, and a daily review of catheter necessity. Process surveillance through the use of a checklist was developed to assess the compliance of four bundle practices and compliance was defined as the frequency of the number of each bundle performed to the number of CVC insertions. Between March 1 and December 31, 2013, the compliance to the CVC insertion bundle was observed. Outcome surveillance, including CLABSI per 1,000 catheter-days, CLABSI per 1,000 inpatient-days, and catheter utilization rates (days of catheter use divided by total inpatient-days), were measured. The data were collected on a routine basis and the analysis was carried out retrospectively. Therefore, informed consent was not required and was specifically waived by the Institution Review Board. Ethics approval was obtained from Institution Review Board of Chi Mei Medical Center.

### Definition

The CLABSI was defined as a primary laboratory confirmed bacteremia or fungemia (excluding skin flora – *Corynebacterium* spp, *Baccilus* spp. *Propionibacterium* spp., coagulase-negative *Staphylococci*, *Streptococcus viridans*, *Aerococcus* spp, *Micrococcus* spp) in a patient with a central line at the time of (or within 48-hours prior to) the onset of symptoms and the infection is not related to an infection from another site [12]. The diagnosis was made jointly by a team of that included the infection control practitioner and intensivists. As a baseline measurement for a comparison, we retrospectively collected the same data from March 1, 2012 to December 31, 2012.

### Statistical analysis

All significant variables in the univariate analyses were included in a logistic-regression model to identify the most important factors associated with the rate of CLABSI. A *P* value < 0.05 was considered statistically significant. The chi-square analysis of the trend was used to assess temporal changes in the rate of infection and catheter utilization. All statistical analyses were conducted using the statistical package SPSS for Windows (Version 19.0, SPSS, Chicago, Il, USA).

## Results

During this 10-month period in the ICU, there were a total of 18,656 inpatient-days and 9,388 catheter-days. The overall catheter utilization rate was 50.3%. Among a total of 687 CVC insertions on 481 patients (134 patients had multiple catheter insertions), 627 (91.2%) insertions were performed by intensivists. Additionally, 38 (5.5%), 12 (1.7%), and 10 (1.5%) CVC insertions were performed by cardiologists, trained residents, and surgeons, respectively. The internal jugular vein was the most common site of CVC insertion (n = 375, 54.6%), followed by the femoral vein (n = 261, 40.0%) and the subclavian vein (n = 51, 7.4%). The overall compliance of all four components of central line insertion bundles was 55.2%. The compliance of each component was as follows: 100% for hand hygiene, 99.6% for the use of CHG, 87.3% for maximal sterile barrier precaution, and 62.2% for optimal site selection.

During this intervention period, there were six CLABSI occurring in six patients, and these infections were diagnosed 7 – 15 days after insertion. The overall rate of CLABSI was 0.64 per 1,000 catheter-days and 0.32 per 1,000 inpatient-days. *Candida* species compromised 4 CLABSI cases, and each one was caused by methicillin-resistant *Staphylococcus aureus* and coagulase-negative *Staphylococcus*. Five CLABSIs were CVC-related and one was double-lumen catheter-related. Three catheters were inserted via the femoral vein, and three central line insertions did not follow the precaution of maximal sterile barrier during insertion. In addition, three infected central lines were inserted by intensivists, followed by a cardiologist (n = 2) and a surgeon (n = 1). The overall compliance of all four insertion bundles of these CLABSI cases was only 33.3%.

Furthermore, we compared the adherence to each CVC insertion bundle between patients with CLABSI and patients without CLABSI (Table 1). We found that CLABSI was more likely to develop in subjects in which a maximal sterile barrier was not used than in subjects in which it had (*P* = 0.03). Moreover, CVCs inserted by non-intensivists were more likely to become infected than CVCs inserted by intensivists (*P* = 0.010). Additionally, CLABSI were more likely in CCU than in MICU and SICU. However, the occurrence of CLABSI was not found to be associated with the type of catheter, site of insertion, hand hygiene, use of 2% CHG, or even the completeness of four insertion bundles. The results of the multivariate analysis disclosed that maximal sterile barrier upon insertion and CVC insertions by intensivists were independently associated with the lower rate of CLABSI. The associated odds ratios (OR) and 95% confidence intervals (CI) are displayed in Table 2.

**Table 1:** Comparison between cases with central line associated bloodstream infection (CLABSI) and cases without CLABSI

**Variables**	**No (%) of cases with CLABSI**	**Univariate**	**Multivariate**
Type of catheter		0.888	
Central venous catheter	5 (0.90)		
Double lumen catheter	1 (0.77)		
Hand hygiene			
Yes	6 (0.87)		
No	0 (0.0)		
Site of insertion		0.679	
Femoral site	3 (2.95)		
Non-femoral site	3 (0.70)		
Use of 2% CHG		0.871	
Yes	6 (0.87)		
No	0 (0.0)		
Maximal sterile barrier		**0.030**	**0.005**
Yes	3 (0.50)		
No	3 (3.45)		
Complete of four bundle		0.416	
Yes	2 (0.53)		
No	4 (0.66)		
Inserted by intensivists		**0.010**	**0.030**
Yes	3 (0.48)		
No	3 (5.00)		
Category		**<0.001**	0.220
Medicine	2 (0.39)		
Surgery	2 (1.37)		
Cardiovascular	2 (7.41)		

Bold type indicates statistical significance with p < 0.05.

**Table 2:** Odds ratio between cases with central line associated bloodstream infection (CLABSI) and cases without CLABSI

	**Odds ratio**	**95% CI**
Maximal sterile barrier (ref: non adherence)	0.141	0.028-0.709
Inserted by intensivists (ref: non-intensivists)	0.091	0.018-0.463

**Figure 1:**
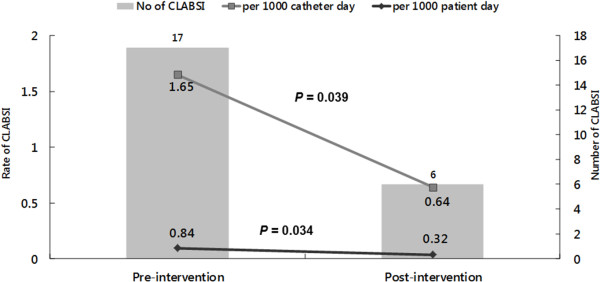
Rate of central line-associated bloodstream infections (CLABSI) and catheter utilization rate during the pre- and post-intervention period.

To further evaluate the effect of the implementation of central line bundle, we compared the rate of CLABSI and catheter utilization between the same 10-months period in 2012 and 2013 (Figure 1). Between March and December in 2012, there was a total of 20,059 inpatient-days and 10,325 catheter-days. The overall catheter utilization rate was 51.4%, and a total of 17 episodes of CLABSI developed during this period (Table 3). We found the rate of CLABSI significantly declined, from 1.65 per 1000 catheter-day during pre-intervention period to 0.64 per 1000 catheter-day post-intervention period (*P* = 0.039). In other words, the rate of CLABSI decreased from 0.84 per 1000 inpatient-days during the pre-intervention period to 0.32 per 1000 catheter-day during the post-intervention period (*P* = 0.034). In contrast, the catheter utilization rate remained stable (*P* = 0.11).

**Table 3:** The number of CLABSI, catheter-day and patient-day per month during the pre- and post-intervention period

**Month**	**3**	**4**	**5**	**6**	**7**	**8**	**9**	**10**	**11**	**12**
Pre-intervention period (2012)										
Catheter-day	867	843	918	954	852	1222	1042	1210	1223	1194
Patient-day	2093	2071	2146	1956	2045	2054	1859	1945	1928	1962
Number of CLABSI	1	1	2	0	1	3	1	0	3	5
Post-intervention period (2013)										
Catheter-day	1138	730	854	742	995	1072	882	871	1098	1006
Patient-day	2019	1821	1754	1738	1967	1882	1832	1846	1957	2140
Number of CLABSI	2	1	1	0	0	0	0	2	0	0

## Discussion

Our investigation in adult ICUs in a regional hospital had several significant findings. First, our findings indicated that the rate of CLABSI can be significantly reduced after implementation of a multidisciplinary quality-improvement intervention, including a central line insertion care bundle. This result is consistent with previous studies [13-19] of different settings (ICU and wards), populations (adult and children), and countries (developed and developing countries). Despite the fact that the bundle care in each study may not be identical, all of these studies suggest that CLABSI can be effectively controlled by applying a multidisciplinary infection control process.

Although we attempted to implement bundle care, including hand hygiene, maximal sterile barriers upon insertion, use of CHG for skin preparation, and avoidance of the femoral vein for the access site in this quality-improvement process, the overall compliance of all four bundles was only 50.3%. The compliance in our study is much lower than a previous study by Osorio et al., in which the staff adherence to the insertion bundle was over 80% in ICUs in Colombia [20]. Moreover, the compliance of the optimal insertion site selection and maximal sterile barrier was only 87% and 62%, respectively. In contrast, the compliance was more than 99% for hand hygiene and use of 2% CHG. It indicated that the process surveillance instigation is warranted to find out the specific deficit of the quality-improvement process. Based on our findings, we should try harder to enhance the adherence to these two CVC insertion bundles, maximal sterile barrier and optimal site selection, in our institution.

To facilitate the maximal sterile barrier, we required that critical care nurses assist with this procedure. Therefore, nurses as well as physicians need to wear masks, sterile gowns, caps, and sterile gloves during CVC insertion, which is labor-intensive and time-consuming. Thus, maximal sterile barrier sometimes cannot be fully applied, especially in the emergency conditions. Finally, the compliance of maximal sterile barrier was low in the present study.

Despite four major components are at the same time included in the central line insertion bundle in the present work, we found the associations between each component and the occurrence of CLABSI were different. Only maximal sterile barrier during insertion was found to be significantly associated with a lower CLABSI rate (OR: 0.141, 95% CI: 0.028-0.709). Our findings and a previous study [10] indicate that this precaution, maximal sterile barrier upon insertion, is essential for the prevention of CLABSI. In contrast, the optimal site of insertion (femoral vein or non-femoral vein access) was not found to be significantly associated with the occurrence of CLABSI. A recent systematic review and meta-analysis [21] by Marik et al. concluded there was no difference in the CLABSI rate with femoral venous catheters compared to subclavian and internal jugular venous catheters in recent studies. In addition, a multicenter randomized trial showed the femoral and internal jugular access had a similar risk of CLABSI while subclavian access in not possible [22]. Therefore, although the avoidance of femoral vein access is recommended as part of central line insertion bundle, the benefit of this measure on the occurrence of CLABSI remains unclear. In summary, our results suggest that maximal sterile barrier during insertion may be the most effective preventive strategy among the four central line insertion bundles.

In addition to bundle care, a CVC inserted by the intensivist was found to be a protective factor for CLABSI in the present work (OR: 0.091, 95% CI: 0.018-0.463). This significance could be explained by the fact that intensivists may have more experience and be more familiar with the insertion of CVC than non-intensivists (77.0% vs 38.1%, p < 0.001). Additionally, our previous study^11^ demonstrated that the performance of CVC insertion bundles was significant better for intensivists than non-intensivist staff (63.9% vs 28.6%, p < 0.001), indicating that CVCs inserted by intensivists in an ICU had the higher compliance of bundle care and lower risk of CLABSI than CVCs inserted by non-intensivists.

Finally, a recent meta-analysis on quality improvement interventions for CLABSI prevention suggested an additional preventive effect through use of bundles/checklists [23]. It reminds that the use of a bundle/checklist with compliance measurement should be another important intervention, and it can identify gaps in prevention measure compliance specific to individual ICUs. Thus, the hospital administrators can develop training programs for those interventions with lower rates of compliance to further enhance the quality-improvement.

This study has several limitations. First, we conducted this investigation at a single institution; therefore, our findings may not be generalizable to other hospitals. The difference between external and internal validity should therefore be more nuanced. In other ICUs the compliance with these care items will differ, so the impact of maximal sterile barrier precautions on the CLABSI rate will be different when there are different maximal sterile barrier compliance rates between hospitals/ICUs. An ICU with 80% maximal sterile barrier use will not benefit as much from a quality improvement bundle encouraging compliance with maximal sterile barrier precautions versus an ICU with 20% compliance. Second, the study was conducted during a short period of time (ten months). Third, we only investigated the impact of the central line insertion bundle on CLABSI but did not evaluate the effect of the central line maintenance bundle. However, the maintenance care of the central line did not have a significant change during the study period. Thus, the impact of maintenance bundle in this study may be minimal. Finally, although a longer catheter duration is a well-known risk factor for CLABSI insertion, these data were not available.

## Conclusions

In summary, this multidisciplinary infection control intervention, including a central line insertion care bundle, can effectively reduce the rate of CLABSI. The impact of different care bundle varies, and a maximal sterile barrier upon insertion is an essential component of the care line insertion bundle.

## Competing interests

All of the authors reported no conflict of interest relevant to this article.

## Authors’ contributions

HJ and HL draft the manuscript, YH, PO, and YC participated in the design of the study, CC drafted and completed the manuscript.

## Pre-publication history

The pre-publication history for this paper can be accessed here:

http://www.biomedcentral.com/1471-2334/14/356/prepub
